# Dimensional stability of cement paste and concrete subject to early-age carbonation curing

**DOI:** 10.1617/s11527-022-01926-8

**Published:** 2022-03-29

**Authors:** Xiangping Xian, Chad Logan, Yixin Shao

**Affiliations:** Department of Civil Engineering, McGill University, 817 Sherbrooke Street West, Montreal, QC H3A 2K6 Canada

**Keywords:** Early age carbonation curing, Dimensional stability, Weathering carbonation

## Abstract

Early-age carbonation curing of concrete is receiving more interest in terms of performance improvement and emission reduction. However, the volume change of cement-based products subject to carbonation curing may become a concern because of the potential carbonation shrinkage and its related shrinkage cracking. The purpose of this study was to investigate the dimensional stability of cement paste and concrete subject to the early-age carbonation curing. It was found that the carbonation curing introduced first an initial shrinkage due to water evaporation upon gas injection and then generated an expansion due to CO_2_ uptake and carbonate precipitation. As carbonation proceeded, the deformation was switched to a secondary shrinkage after expansion. The residual deformation due to carbonation curing was shrinkage in cement paste samples and expansion in concrete samples. This was because the relative expansion due to carbonate precipitation in paste was not large enough to compensate for the shrinkage caused by water loss. However, for concrete samples, the introduction of aggregates reduced the pore spaces in concrete, leading to an expansion owing to the limited precipitation. The results of carbon dioxide uptake, XRD, and SEM analysis confirmed that calcium carbonate formation played a critical role in the relative expansion. The study also showed that cement-based products were more resistant to weathering carbonation after the early-age carbonation curing. After 61-day weathering carbonation exposure, both paste and concrete samples exhibited carbonation shrinkage as a result of carbonation of hydration products. However, the magnitude of shrinkage was much smaller in carbonation curing than in weathering carbonation because of the short period of exposure. Both carbonations did not significantly affect the compressive strength of carbonated products. Carbonation curing likely makes concrete products more dimensionally stable in the long-term service.

## Introduction

Early-age carbonation curing is developed for accelerated strength gain for precast cement-based products that require fast curing [1–3]. It is also found that once treated by early carbonation, concrete masonry blocks have shown more resistance to atmospheric carbonation in service and to its related shrinkage-induced cracking [4]. The drying shrinkage caused by cycles of wetting and drying is also reduced. Carbonation curing treatments have been reported to enhance surface hardness and decrease permeability and have been used as a method to improve the frost and surface wear resistance, as well as the resistance to alkali-aggregate reaction [5]. Owing to the denser surface created by carbonation curing, the concrete demonstrates higher surface resistivity [6] and presents improved resistances to freeze-thaw cycles [7], sulfate attack [8], efflorescence formation [9], and chloride penetration [10]. Besides, early carbonation does not increase the corrosion potential of steel in reinforced concrete because of the low degree of carbonation [11, 12].

Early carbonation curing is substantially different from service weathering carbonation in that the former reaction occurs with calcium silicate compounds immediately after concrete formation [1], while the latter happens to react with hydration products throughout the entire service life [13]. Service weathering carbonation decomposes hydration products, making concrete more vulnerable to carbonation shrinkage. Extensive studies have been carried out to understand the mechanism and develop the corrective measurements. One hypothesis is proposed by Powers that calcium hydroxide dissolves into the water phase before reacting with dissolved carbon dioxide (carbonic acid) to generate calcium carbonate [14]. The primary reaction of service weathering carbonation is between calcium hydroxide and carbon dioxide, even though several other hydration products react with carbon dioxide as well, to produce calcium carbonate. Based on Powers’s theory, carbonation shrinkage is attributed to the temporary increase in compressibility of paste resulting from the dissolution of calcium hydroxide from areas under compressive stress, so the decalcification of C–S–H is considered as less significant. The other hypothesis has also been proposed that the dehydration of C–S–H by weathering carbonation will cause carbonation shrinkage [15]. A recent study postulates that carbonation shrinkage due to the carbonation of C–S–H manifests the general phenomenon of decalcification shrinkage [16]. Studies speculate that the carbonation of calcium hydroxide and C–S–H are simultaneously reacting [17, 18]. Ramachandran and Feldman put forward that the van der Waals’ surface forces are the reasons for inducing carbonation shrinkage. As calcium hydroxide is dissolved away between points of contact, the van der Waals’ forces pull the crystals together. This hypothesis also indicates that the carbonation of C–S–H makes a contribution to shrinkage by silica polymerization [19]. The volume change of cement-based materials subject to weathering carbonation always presents dissolution-induced shrinkage while the crystallization-induced expansion after carbonation is also found in the OPC paste containing alkali salts due to the extensive generation of more calcite [20].

Recently, early carbonation has gained renewed interest. Since carbonation is a carbon uptake process, it can be utilized by the precast concrete industry to permanently store carbon dioxide in a beneficial manner [21, 22]. Carbonation curing has been successfully applied in various precast products including concrete masonry unit [23], eco-concrete [24], steel slag block [25], and even reinforced concrete [26]. The carbon cap and trade system for emissions reduction in the near future will produce a large quantity of carbon dioxide that could be affordable and available for the carbonation process. Furthermore, directly using flue gas is also applicable for a carbonation curing system [27, 28]. This will definitely provide incentives for concrete producers to consider the carbonation process for carbon credits. To promote the integration of carbon sequestration and more applications into concrete production, the dimensional stability of concrete products subject to early carbonation curing has to be examined due to the carbonation shrinkage at early age.

Volume change due to early carbonation curing is not well studied. Because of the exothermic carbonation reaction, change in dimension in fresh state of cement and concrete will occur and could have a significant influence on their structural integrity. The purpose of this paper is to examine the in-situ dimensional change of cement and concrete subject to early-age carbonation curing and the concrete resistance to service weathering carbonation after early-age carbonation curing. Cement paste samples were applied to attain the most drastic results while concrete samples were adopted to simulate concrete products. Two types of early carbonation were studied. One was applied immediately after concrete formation and the other was applied after 17-h pre-setting hydration. The influences of early carbonation reaction on carbon uptake and strength development at different ages were also discussed. X-ray diffraction (XRD) and scanning electron microscopy (SEM) were also used to characterize the carbonation products after early-age carbonation curing.

## Experimental program

### Sample preparation

The mixture proportion of cement, water, river sand and limestone coarse aggregate is shown in Table 1. General Use (GU) Portland cement (CSA-3001) produced by Lafarge Canada was used with a CO_2_ content of 0.54% and Blaine fineness of 373 m^2^/kg. River sand had a fineness modulus of 2.3 and the maximum aggregate size of limestone was 6.5 mm. Cement paste was adopted to quantify carbon uptake without the interference of coarse limestone aggregates while concrete was a special dry mix concrete to simulate precast products such as blocks, paving stones, pipes, and hollow-core slabs. The constituents were immediately mixed for approximately 5 min using an industrial pan mixer. Samples were individually compact formed under a pressure of 8 MPa and then stored in a sealed container until all samples in one batch were made. Two types of sample geometries were employed: rectangular slabs (76 × 127 × 19 mm) for CO_2_ uptake and strength evaluation and square bars (25 × 25 × 279 mm) for volume change measurement. Batches 1, 3, 5 and 7 were early carbonation-treated immediately after compact casting while batches 2, 4, 6, and 8 underwent a 17-h pre-setting hydration before the early-age carbonation. Carbonation duration varied from 2 to 18 h, as the carbonation pressure was maintained constant at 500 kPa. The process parameters are also summarized in Table 1. For one batch, at least eighteen slabs (6 tested immediate after carbonation, 6 tested after carbonation and 7d hydration, and 6 tested for weathering carbonation after 7d hydration) and seven bars (1 bar tested for in-situ volume change by carbonation strain measurement, 6 tested for volume change due to weathering carbonation) were prepared by the same procedure. Of the 6 samples tested at one time, 3 were carbonation cured and 3 hydration cured as reference.

**Table 1:** Mixture proportion and process parameters

Batch	Classification	C	W/C	S/C	CA/C	Preset, hr	Carbonation time, hr
B1	Cement paste	1	0.15	0	0	0	2
B2	Cement paste	1	0.15	0	0	17	2
B3	Cement paste	1	0.15	0	0	0	18
B4	Cement paste	1	0.15	0	0	17	18
B5	Concrete	1	0.26	1.3	2.6	0	2
B6	Concrete	1	0.26	1.3	2.6	17	2
B7	Concrete	1	0.26	1.3	2.6	0	18
B8	Concrete	1	0.26	1.3	2.6	17	18

*C* Cement, *W* Water, *S* River sand, *CA* Coarse limestone aggregate

### Carbonation curing

**Fig. 1:**
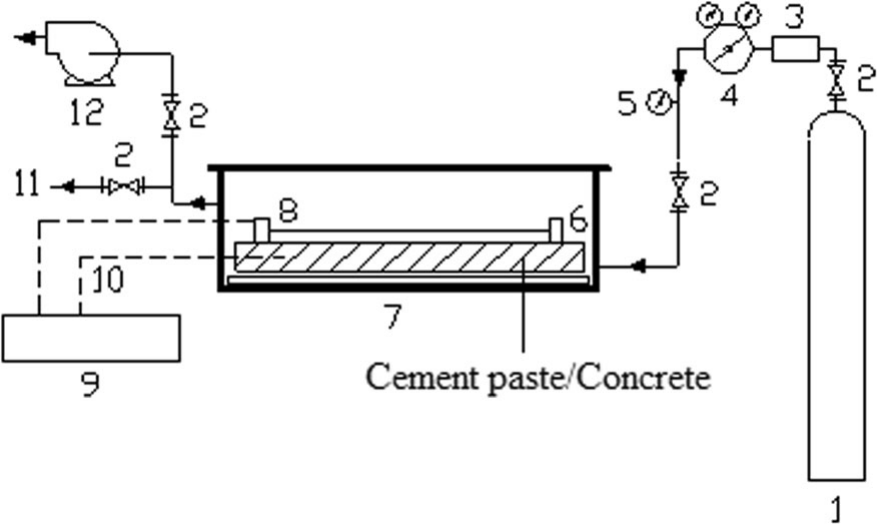
Schematic of carbonation curing apparatus (1—CO_2_ tank, 2—Valve, 3—Heater, 4—Regulator, 5—Pressure gauge, 6—Bar sample, 7—Pressure vessel, 8—LVDT assembly, 9—Data acquisition system, 10—Thermocouple, 11—Discharge, 12—Vacuum pump)

The carbonation curing apparatus is shown in Fig. 1, including a compressed carbon dioxide gas tank, curing chamber, thermocouple, linear variable displacement transducer (LVDT), data acquisition unit and vacuum pump. CO_2_ gas of 99.8% purity was used to simulate recovered carbon dioxide from flue gas sources.

Immediately after curing, water evaporated from exothermic carbonation reaction was then collected from the chamber walls using absorbent paper and was added to the final mass because carbonation process was treated as a closed system. CO_2_ uptake is calculated by percent mass gain between final mass and initial mass as shown in Eq. (1).

1 $${\rm{CO}}_{{\rm{2}}} {\mkern 1mu} {\mkern 1mu} {\mkern 1mu} ~{\rm{uptake}}~\left( \% \right)\;{\rm{ = }}~\;\frac{{{\rm{Mass}}_{{{\rm{final}}}} {\rm{ + }}~{\rm{Mass}}_{{{\rm{water - loss}}}} {\rm{ - Mass}}_{{{\rm{initial}}}} }}{{{\rm{Mass}}_{{{\rm{dry - binder}}}} }}~\;{\rm{ = }}\;~\frac{{\Delta {\rm{Mass}}_{{{\rm{CO2}}}} }}{{{\rm{Mass}}_{{{\rm{dry - binder}}}} }}$$

The validity of using mass gain as a way of quantifying carbon uptake was examined in the tests of carbon content using infrared based carbon analyzer (ELTRA CS800). The powder of cement binder in both cement paste and concrete samples was collected using a diamond drill of 2 mm. The CO_2_ content of paste was examined both on the surface (1 mm deep) and in the core. Since it was difficult to obtain the powder of paste in concrete due to the low cement content, the same drill was used to collect enough binder powder both on the surface and at the core. The mixture of the two served as cement binder in concrete for analysis. To minimize the influence of limestone aggregates on sampling, the same procedure was applied to reference hydrated concrete. The CO_2_ content of concrete due to carbonation curing was estimated by the difference between carbonated and hydrated samples.

### Accelerated weathering carbonation test

**Fig. 2:**
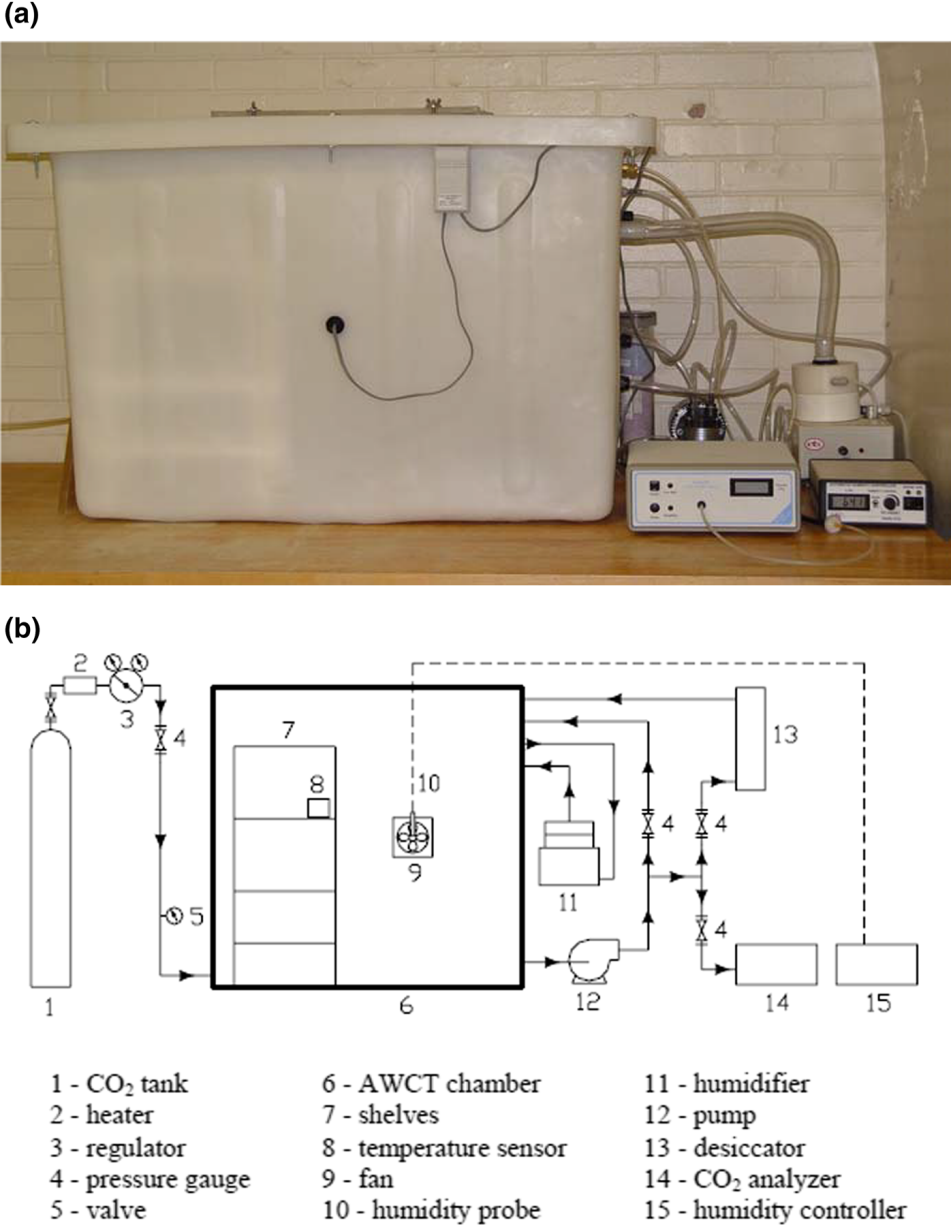
Accelerated weathering carbonation test: **a** Weathering carbonation apparatus; **b** Schematic of weathering carbonation apparatus

To investigate the influence of early carbonation curing on carbonation shrinkage in service, cement paste and concrete samples were exposed to accelerated weathering carbonation testing (AWCT) to simulate the conditions of long-term exposure to atmospheric carbon dioxide during service. The set-up details are displayed in Fig. 2. Following a 7-day hydration in a moisture chamber, both carbonated and hydrated slab and bar samples were subjected to accelerated weathering carbonation testing (AWCT). First, the mass of each sample was determined and the length of bar samples was measured using the DEMEC strain gauge. Samples were then placed in the AWCT chamber. Afterward, carbon dioxide was injected into the chamber to reach a concentration of 50% and the relative humidity of the chamber was set to 65%. Length and mass measurements were taken once every day in the first week, then reduced to once every two days in the following four weeks and once every week in the remaining test. During every measurement, the chamber carbon dioxide concentration, relative humidity and temperature were monitored. The length and mass of each bar sample were individually recorded after a reading was measured from the Invar reference bar. The next step was to measure the mass of each slab sample. After each stage of measurements, the chamber was sealed shut and carbon dioxide was reinjected to have a concentration of 50% while the automatic humidity control was set to the required relative humidity.

### Volume change measurement

Both cement paste and concrete samples were examined for their volume change upon exposure to early carbonation and at their service lives during the accelerated weathering carbonation test. Of the seven designated bars, one was used for in-situ volume change measurement, six were for volume change subject to accelerated weathering carbonation including three treated with early carbonation and three references with only moisture curing.

To quantify the in-situ volume change due to carbonation curing, the LVDT assembly was fixed on two plexiglass mounts which were attached to the sample using epoxy at a gage length of 203 mm. Before carbonation, a small hole was drilled in the side of one bar sample for the thermocouple. Then the mass of each sample including LVDT assembly was recorded as the mass prior to carbonation before placing them in the carbonation curing. Upon the required carbonation period had reached, the carbon dioxide was released into a water tank. During the carbonation period, the deformation and temperature were recorded by System 5100 Scanner every second in the first hour and every 30 s afterward until the designated carbonation time (2 h or 18 h).

To measure the volume change due to weathering carbonation, a demountable mechanical strain gauge (DEMEC strain gauge) was used for a period of 61-day exposure. The stainless steel discs approximately 5 mm in diameter were mounted on the bar samples using epoxy. Two discs were attached 203 mm apart center to center down the middle of the bar as gauge length for strain measurement. The holes in the center of the discs were used by DEMEC strain gauge to obtain a length change accurate to 0.00254 mm.

### Compressive strength

Compressive strength of slab samples for each batch was obtained immediately after carbonation treatment, after subsequent 7-day moisture curing and after subsequent 61-day accelerated weathering exposure. To make a comparison, hydrated slab samples without carbonation were also tested as reference at exactly the same age as carbonated samples. Three identical samples were tested for average.

### X-ray diffraction

In order to study the crystallographic texture of early carbonated samples, X-ray diffraction (XRD) was performed using a Phillips PW1710 Powder Diffractometer with Cu Kα radiation. The scanning patterns were conducted at a 2*θ* from 5 to 60° and a 0.02° step with 0.5 s per step. Same powder samples as used for carbon dioxide content analysis by carbon analyzer were adopted.

### Scanning electron microscopy

To further investigate the effect of early carbonation curing on the microstructure of carbonates, a JEOL JSM-840A equipped with an EDAX Phoenix Energy Dispersive X-Ray Spectrometer (EDS) microanalysis system was employed to conduct the scanning electron microscopy (SEM) test for carbonation-cured samples.

Photomicrographs and EDS scans were also attained for selected samples to compare the chemical composition as well as the crystal structure. The tested samples were collected from the fractured surface after compressive strength testing and stored in alcohol to prevent further hydration and carbonation. Before SEM test, the samples were removed from alcohol, dried, mounted and finally sputter coated with gold to prevent charging.

## Results and discussion

### Early-age carbonation-induced volume change

**Fig. 3:**
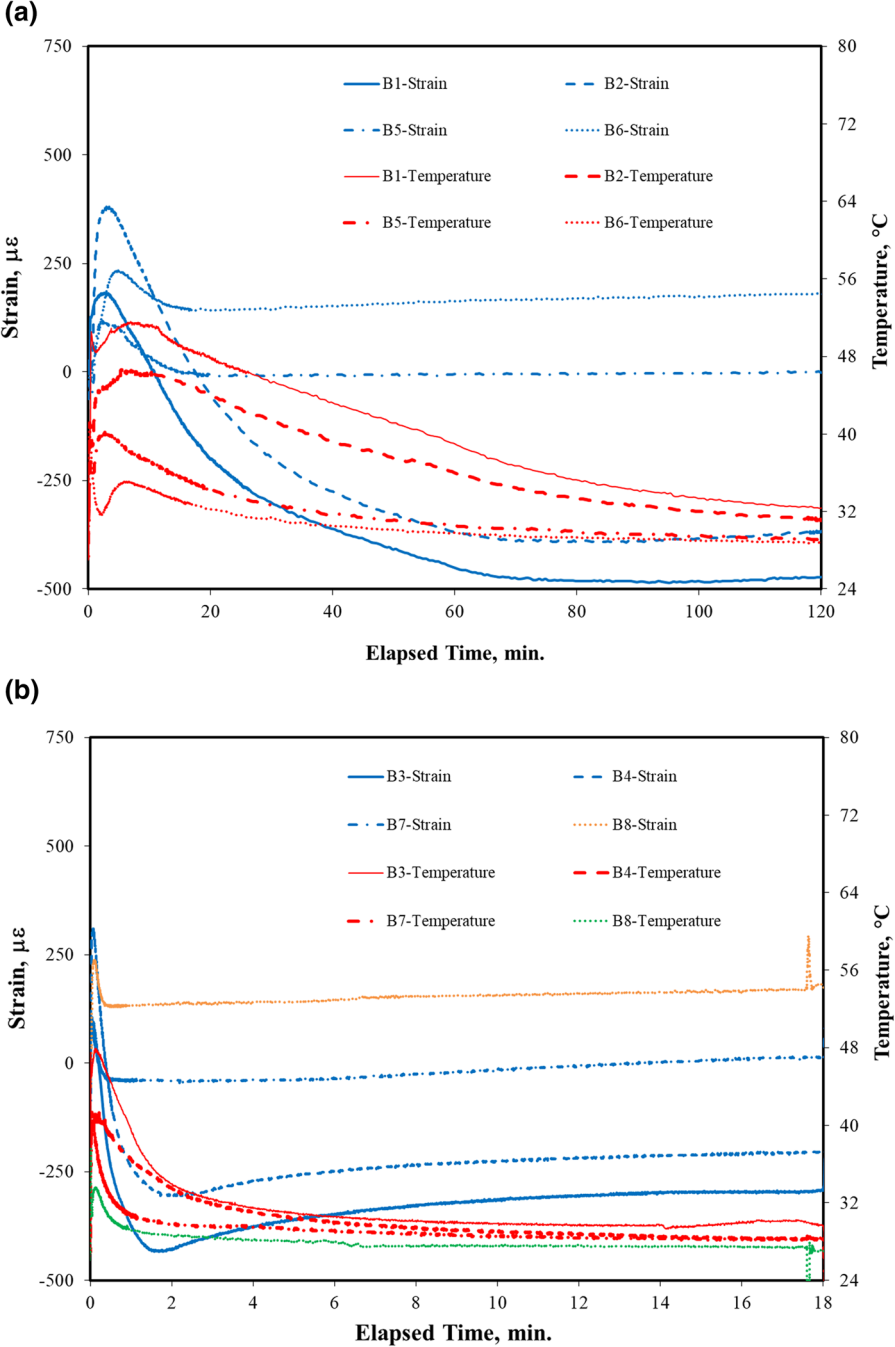
In-situ carbonation strain curves for cement pastes and concrete subject to carbonation curing: **a** 2-h carbonation (B1: Cement paste without preset; B2: Cement paste with 17-h preset; B5: Concrete without preset; B6: Concrete with 17-h preset); **b** 18-h carbonation (B3: Cement paste without preset; B4: Cement paste with 17-h preset; B7: Concrete without preset; B8: Concrete with 17-h preset)

The in-situ strain curves measured by LVDT during carbonation curing for both cement paste and concrete samples are demonstrated in Fig. 3 with corresponding temperature curves. The results of dimensional change are summarized in Table 2. The temperature seemed to correlate with strain measurement from the beginning of carbonation. The deformation pattern was observed in both pastes and concretes. The 25-mm bar sample experienced an initial fast shrinkage immediately after the gas injection. The shrinkage was accompanied by water evaporation and happened in the first one minute. At the same time, the reaction temperature reached a peak. It was then followed by an expansion due to the CO_2_ uptake and carbonate precipitation. After the maximum expansion, the bar went through a secondary shrinkage until the final strain. The secondary shrinkage is defined by the strain difference between the maximum expansion and the final strain. As the carbonation rate decreased and the heat dissipated, the carbonation strain began to reduce, leading to a final deformation as a shrinkage in pastes and an expansion in concretes.

**Table 2:** Carbonation strains induced by early-age carbonation curing

Batch	Carbonation curing	Water loss (%)	Initial shrinkage με	Max expansion με	Final strain με	Secondary shrinkage με
B1	2hC	14.0	− 20	182	− 444	− 626
B2	17hP + 2hC	5.7	− 40	380	− 369	− 749
B3	18hC	14.4	− 50	106	− 289	− 395
B4	17hP + 18hC	3.2	− 50	310	− 225	− 535
B5	2hC	7.9	− 51	118	15	− 103
B6	17hP + 2hC	0.7	− 26	234	165	− 69
B7	18hC	6.3	− 87	96	28	− 68
B8	17hP + 18hC	0.6	− 56	240	172	− 68

1, 17hP:17-h preset, 2hC: 2-h carbonation; 2, Secondary shrinkage = Final strain − Max expansion

In 2-h carbonation of cement paste without preset (B1 in Fig. 3a), the dimensional change started with an initial shrinkage of − 20 με, followed by an expansion with a maximum strain of 182 με in first 10 min. During the expansion period, the temperature reached the peak of 50 °C because of the exothermic carbonation reaction. The expansion was attributed to the calcium carbonate precipitation. The deformation was gradually switched to a shrinkage with a final strain of − 444 με at 2 h. The secondary shrinkage was − 626 με for paste subject to 2-h carbonation without presetting. The shrinkage was accompanied by a 14% water loss due to carbonation (Table 2) and reached a plateau at 60 min. The reaction temperature was reduced accordingly.

For paste with 17-h preset (B2 in Fig. 3a), the initial shrinkage was − 40 με followed by an expansion with a maximum strain of 380 με in 10 min, doubled that of paste without preset. The expansion was then gradually reduced to a shrinkage of − 369 με at 2 h with a water loss of 5.7% (Table 2). The maximum temperature was 46 °C and the secondary shrinkage was − 749 με. It seemed that preset had promoted more carbonate precipitation at the beginning of the reaction and generated less water evaporation in the entire process.

For prolonged carbonation of 18 h, the first 2-h reaction was similar to the 2-h carbonation results. The initial shrinkage was about − 50 με in first 1 min. The maximum expansion happened in first 10 min, reaching 106 με for paste without preset (Fig. 3c) and 310 με for paste with 17-h preset (Fig. 3d). It was followed by a secondary shrinkage with a maximum value of − 395 με without preset and -535 με with preset. The corresponding water loss was 14.4% and 3.2% (Table 2) respectively. Preset of 17-h helped reduce the water loss and its related shrinkage. After the first 2 h, the pastes experienced an expansion at a much lower rate, making the overall deformation by 18-h carbonation a shrinkage of − 289 με for paste without preset and − 225 με for paste with preset.

A similar deformation pattern was observed in concrete subject to carbonation curing. For 2-h carbonation without preset (B5 in Fig. 3e), the concrete experienced an initial shrinkage of − 51 με in first 1 min, followed by an expansion with a maximum strain of 118 με in first 10 min and then a secondary shrinkage with a strain of − 103 με. The maximum reaction temperature was 40 °C, the overall deformation after 2-h carbonation was 15 με with a water loss of 7.9%. It was apparent that concrete underwent a less dimensional change due to less water loss in reaction in comparison with cement pastes. For 2-h carbonation with 17-h preset (Fig. 3f), the initial shrinkage was − 26 με in first 1 min, followed by an expansion of 234 με. The final deformation after 2-h carbonation was an expansion with an overall strain of 165 με and a water loss of 0.7% (Table 2). Again, a preset of 17-h made concrete more reactive in the first 10 min and more resistant to shrinkage during the curing process.

For prolonged 18-h carbonation, the initial shrinkage was − 87 με without preset (B7) and − 56 με with preset (B8) in first 1 min. The subsequent expansion was accompanied by a maximum strain of 96 με for concrete without preset (Fig. 3g) and 240 με with 17-h preset (Fig. 3h). Unlike the cement pastes, the final deformation of concrete subject to 18-h carbonation was an expansion with an overall strain of 28 με without preset and 172 με with preset. The overall water loss in 18-h carbonation was 6.3% without preset and 0.6% with preset (Table 2), similar to 2-h carbonation. It seemed that most reactions occurred in the first 2 h.

Table 2 also shows the effect of carbonation duration, preset treatment, and material types (cement/concrete) on the volume change of samples. Increasing carbonation duration from 2 to 18 h did not increase more dimensional change. Instead, there was a reduction in maximum expansion and a reduction in secondary shrinkage in pastes after 18-h carbonation. Longer carbonation of 18-h in B3 could help cement paste to have less shrinkage than 2-h carbonation in B1 by comparing their final strain values, reading − 289 με versus − 444 με. Specifically, the strain difference due to the additional carbonation of 16 h between them was 155 με, which could be considered the reduction of shrinkage between these two batches. This was also true when including preset treatment for pastes in comparison of final strains between B2 and B4, whose strain difference was 144 με. All paste samples presented the final status of volume change as shrinkage since the relative expansion due to carbonate precipitation as shown in Fig. 3 could not compensate for the shrinkage caused by water loss. However, for concrete with aggregates, all batches presented expansion as their final deformation after early-age carbonation curing. Longer carbonation created more expansion in concrete. This curing-induced expansion will be beneficial to structural resistance to drying shrinkage in service.

The preset treatment by 17-h hydration before carbonation could also lead to more expansion compared to those without preset. For the same carbonation duration, 17-h preset before carbonation for paste B2 and B4 had smaller final shrinkage than that without preset treatment in paste B1 and B3. The expansion effect was more distinct in the carbonation of preset concretes. After a 17-h preset, the final strain of 2-h carbonation (B6) could be increased more than 10 times than that without preset treatment (B5), but this pronounced result was reduced to 6 times when making a comparison between B7 and B8 due to longer carbonation. It seemed carbonation of both calcium silicates and calcium hydroxide might produce more calcium carbonates, thus causing more CO_2_ uptake and more expansion after their deposition.

In Table 2, the secondary shrinkage as absolute volume change was calculated to obtain the overall dimensional change under carbonation curing. The absolute volume change was defined as relative shrinkage between the maximum expansion and the final overall strain. This was the shrinkage generated by carbonation curing and could lead to shrinkage cracking if the threshold value was exceeded. For pastes, the average of absolute volume change was 688 με for 2-h carbonation (B1 and B2) and 465 με for 18-h carbonation (B3 and B4). For concrete, the absolute volume change was much lower. It was 86 με for 2-h carbonation (B5 and B6) and 68 με for 18-h carbonation (B7 and B8). Less absolute volume change indicated higher resistance to deformation and lower chance of shrinkage cracking. Therefore, carbonation curing of concrete up to 18 h will not generate significant shrinkage which may lead to cracking in concrete under a restrained condition.

### Carbon dioxide uptake

Table 3 shows the CO_2_ uptake of cement paste samples subject to carbonation curing. It was calculated by the mass gain method and carbon analyzer method. The mass gain method was based on Eq. 1 and was averaged throughout the entire sample. It was independent from the initial carbon content. CO_2_ uptake by the mass gain method was obtained from both bar specimens and slab specimens. CO_2_ content was measured by a carbon analyzer from the surface and the core of slab specimens. They were in the same order of magnitude. The averages of the three measurements are also presented. For the 2-h carbonation of cement pastes, the average CO_2_ uptake was 10% based on cement mass. For 18-h carbonation, it was 12.7%. Longer carbonation duration could enhance the carbon dioxide uptake in the paste. However, this increase in uptake was not proportional to the increase in carbonation duration. This was possibly due to the fact that rapid carbonation could create carbonate build-up on the surface, blocking further penetration of carbon dioxide [2, 29]. On the other hand, 17-h preset treatment seemed to have little effect on uptake. It was noted that presetting by hydration before carbonation could generate more hydration products including calcium hydroxide and calcium silicate hydrate so that more water was bound in the hydration products. Much less water loss was detected in preset samples. Therefore, there was less free water in the pore structure to promote carbonation.

**Table 3:** CO_2_ uptake of cement paste samples subject to carbonation curing

Batch	Uptake, % (bar)	Uptake, % (slab)	CO_2_ content, % (slab)			Average CO_2_ uptake, %
			Surface	Core	Ave	
B1	10.65	10.83	10.21	8.83	9.52	10.33
B2	9.58	10.85	9.52	8.95	9.24	9.89
B3	13.78	12.68	11.70	10.08	10.89	12.45
B4	13.29	13.22	13.46	12.04	12.75	13.08

1, CO_2_ uptake based on Eq. 1; 2, CO_2_ content: Measured by CO_2_ analyzer

Table 4 displays the CO_2_ uptake of concrete samples subject to carbonation curing. Similar to cement paste, both concrete bar samples and concrete slab samples were used. The three measurements were comparable and averaged for comparison. In terms of CO_2_ uptake, concrete had shown smaller uptake than paste for the same carbonation duration or preset treatment. This was probably owing to the lower cement content in concrete samples. For 2-h carbonation, the average of CO_2_ uptake by concrete was 8.56%. For 18-h carbonation, it was 12.3%. Longer carbonation duration promoted more CO_2_ uptake. Nevertheless, preset treatment had less CO_2_ uptake. After preset treatment concrete had higher maximum expansion strains right at the beginning of carbonation. Hydration products were more susceptible to be influenced by the exothermic reaction of carbonation. Besides, concrete samples after 17-h preset treatment became denser and had less free space for carbonate precipitation. The water losses were also lower, recording only 0.7% and 0.6%, respectively. The less free water in the pore structure was the possible reason for the lower CO_2_ uptake for presetting samples of B6 and B8. It was said that optimal carbonation was restrained when the relative humidity was below 50% [30]. The surface area of free water in pores was much smaller under low relative humidity, dramatically slowing the dissolution of CO_2_ and the subsequent carbonation reaction.

**Table 4:** CO_2_ uptake of concrete samples subject to carbonation curing

Batch	Uptake, % (bar)	Uptake, % (slab)	CO_2_ content, % (slab)			Average CO_2_ uptake, %
			Carb	Hyd	Diff	
B5	9.70	10.15	15.22	7.22	8.0	9.28
B6	7.38	8.37	16.10	8.36	7.74	7.83
B7	12.98	15.02	20.39	7.52	12.87	13.6
B8	11.75	10.46	20.11	9.25	10.86	11.0

1, CO_2_ uptake based on Eq. 1; 2, CO_2_ content: Measured by CO_2_ analyzer; 3, Carb: Carbonated; Hyd: 4, Hydrated; Diff: Difference (CO_2_ content due to carbonation)

### X-ray diffraction of selected cement paste samples

**Fig. 4:**
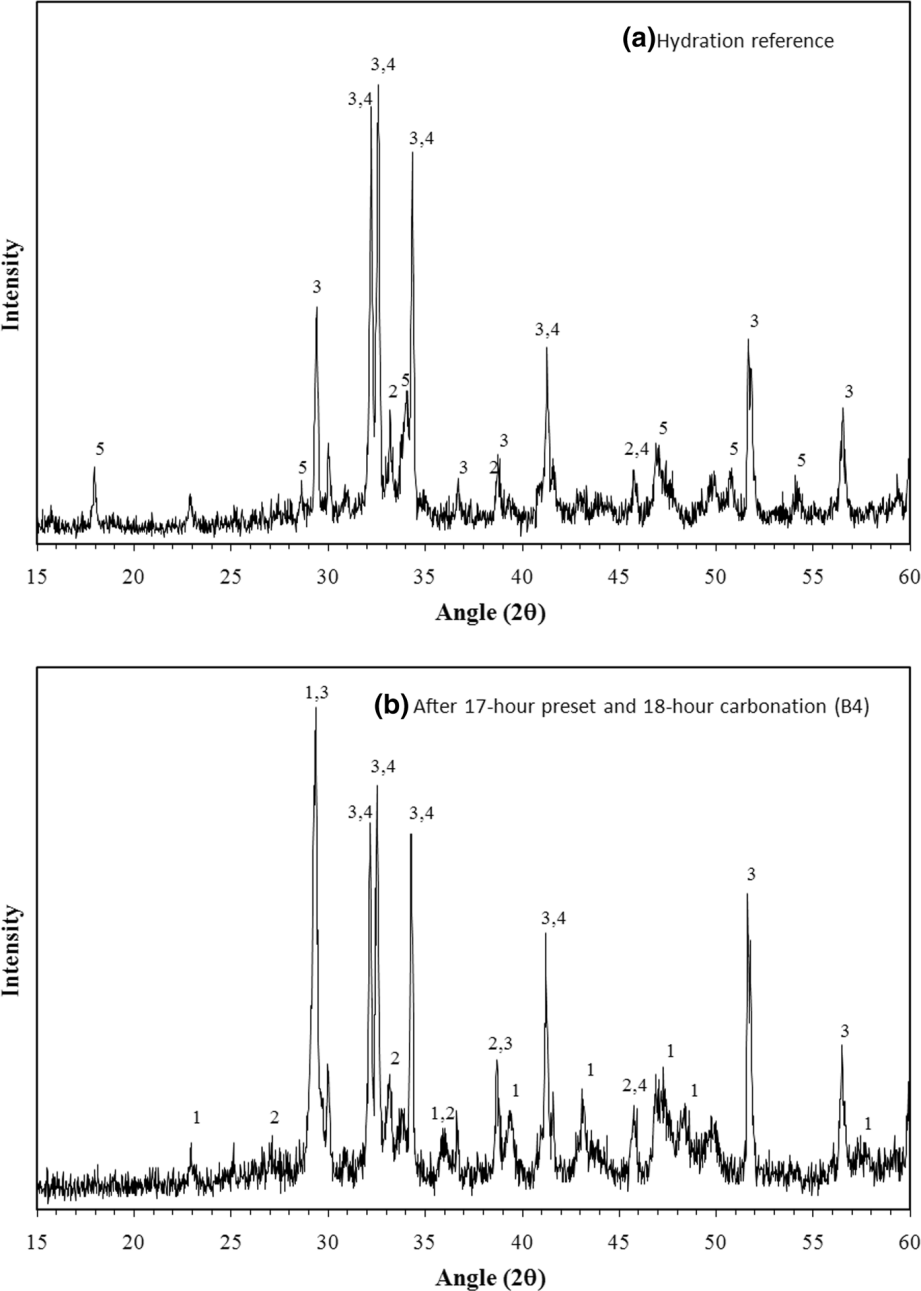
XRD Analysis of cement paste samples: (1) calcite, (2) aragonite, (3) C_3_S, (4) C_2_S, (5) calcium hydroxide: **a** Surface layer of hydrated cement paste; **b** Surface layer of carbonated cement paste after preset

Figure 4 shows the X-ray diffraction analysis of selected samples. Figure 4a was the XRD pattern for normal hydrated reference paste while Fig. 4b was that for early carbonated paste. After 17-h preset treatment and 18-h carbonation, B4 paste sample presented the most CO_2_ content at the surface layer using infrared-based carbon analyzer. It was noted that calcium carbonates in form of calcite and aragonite were detected in B4 after carbonation in comparison of XRD patterns between hydrated and carbonated samples. The carbonate precipitation during carbonation curing was the primary reason for B4 to have higher resistance to absolute volume change thus having the least final shrinkage of − 225 με as compared to other paste results in Table 2. Relatively high and intense peaks of C_3_S and C_2_S in carbonated paste B4 indicated that unreacted cement grains still existed even after 18-h carbonation, which could be reactive during subsequent hydration to further enhance the sample resistance to volume change. No significant evidence of calcium hydroxide in the carbonated sample suggested that calcium hydroxide was the most reactive material to react with carbon dioxide. Furthermore, the consumption of calcium hydroxide could also possibly contribute to the concrete resistance to volume change due to the weathering carbonation reaction.

### Scanning electron microscopy of selected samples

**Fig. 5:**
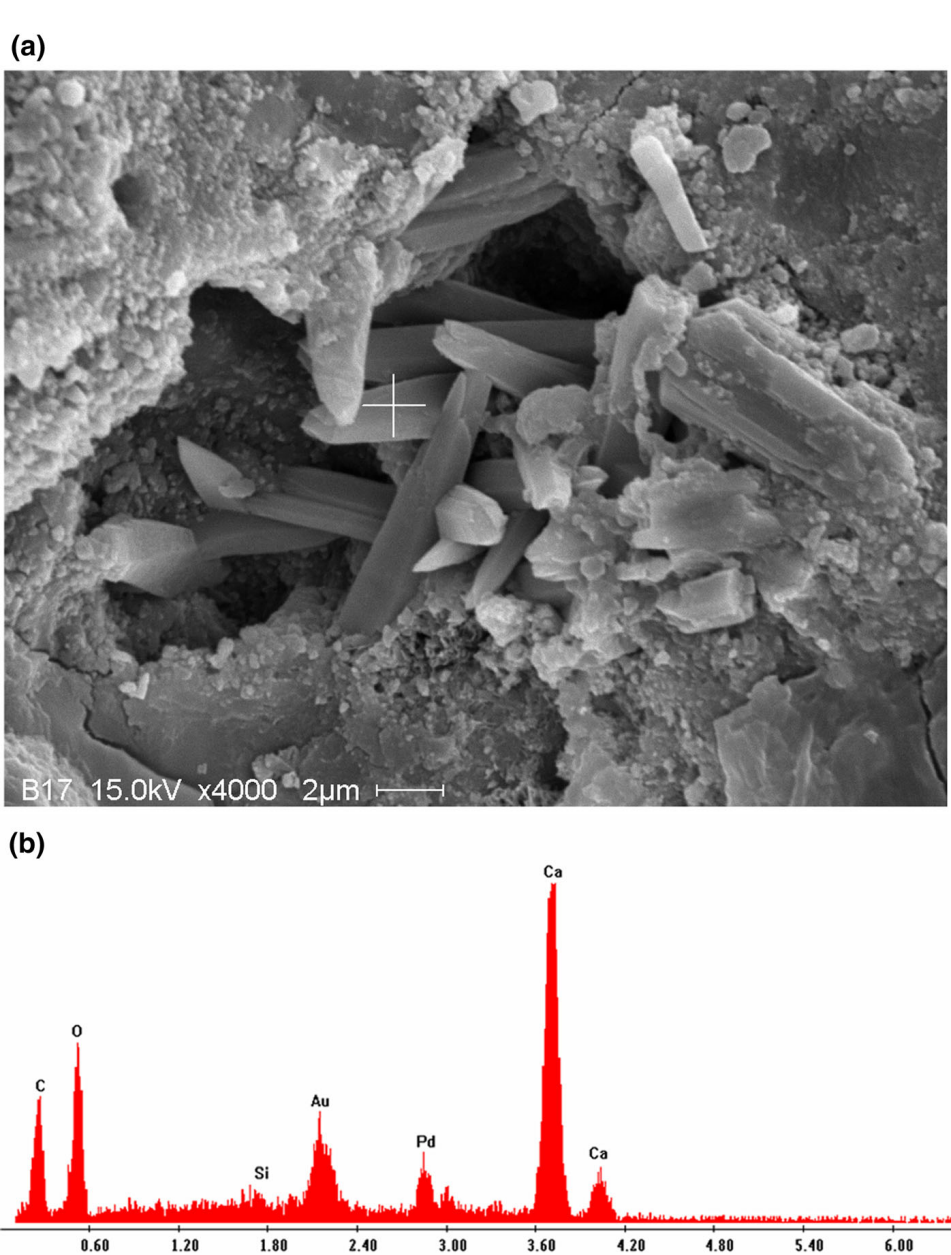
Microstructure of cement paste after 18-h carbonation curing: **a** SEM micrograph; **b** EDS pattern

**Fig. 6:**
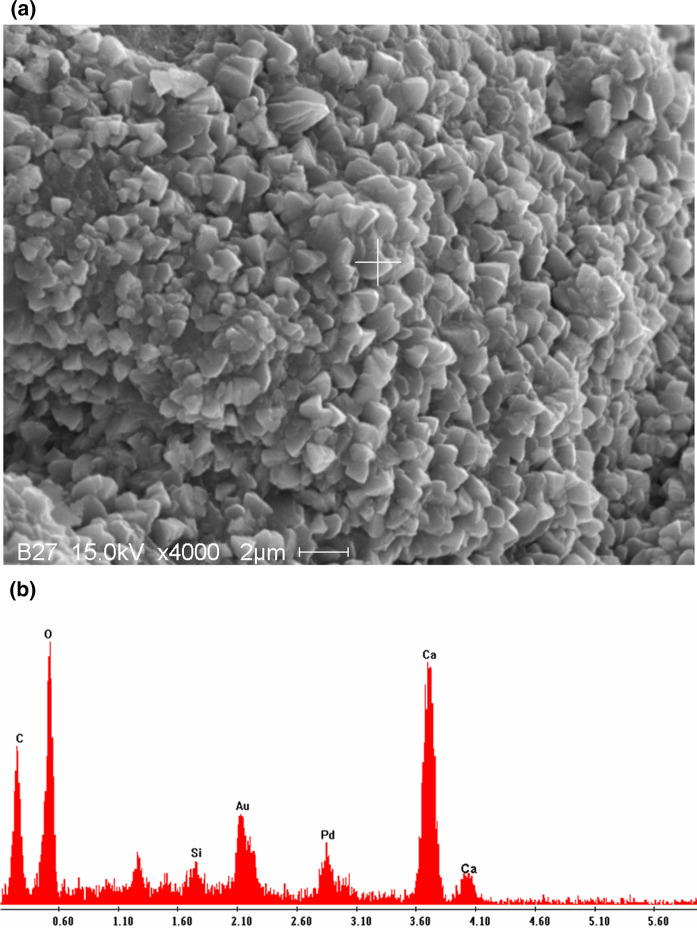
Microstructure of concrete after 18-h carbonation curing: **a** SEM micrograph; **b** EDS pattern

Figure 5 displays the morphology of calcium carbonate at the paste surface layer after 18-h carbonation curing at a magnification of × 4000. The pointed-out prismatic crystal could be aragonite generated during early carbonation, filling in the pore structures since its EDS point analysis contained amounts of calcium, carbon and oxygen. This indicated that carbonated paste after 18-h carbonation could have more resistance to volume change due to the carbonate precipitation. The conclusion was also true and more remarkable for the observation of early carbonated concrete sample, shown in Fig. 6. Unlike cement paste in which more pore spaces were produced by hydration, the concrete sample presented a much denser structure. Figure 6a clearly presented a large quantity of small carbonates densely formed in the pore spaces. The introduction of aggregates reduced the pore spaces in concrete, leading to a more crowded precipitation of carbonates (seemingly calcite which was more voluminal), generating large crystallization stress and afterward resulting in a limited expansion of the whole sample [20]. This could explain the final expansion status of concrete samples after carbonation as shown in Table 2. However, since the precipitated volume of carbonates in the cement paste sample did not exceed that due to the cement hydration, all early carbonated paste samples demonstrated their final volume changes as shrinkage status.

**Fig. 7:**
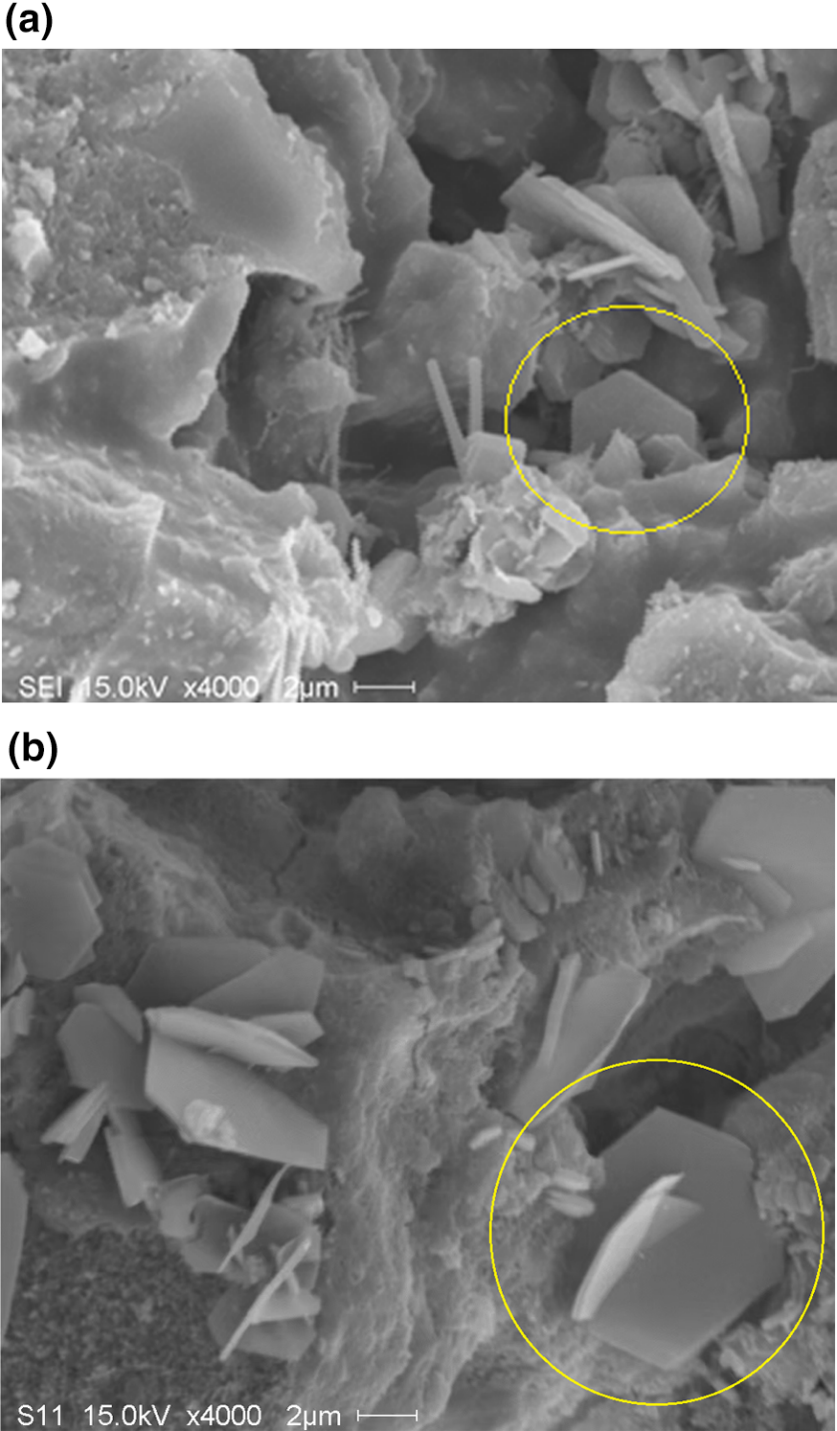
Microstructure of cement paste and concrete after 18-h hydration: **a** SEM micrograph of hydrated cement paste sample; **b** SEM micrograph of hydrated concrete sample

Figure 7a shows an SEM micrograph of hydrated cement paste from the surface layer. In contrast to the carbonation cured sample, the growth of hexagonally shaped crystal was found as indicated within the yellow circle, which was potential evidence of calcium hydroxide being produced during hydration. The existence of hexagonal calcium hydroxide was more distinct in the hydrated concrete sample as shown in Fig. 7b. Conclusively, calcium hydroxide was consumed during carbonation curing to generate calcium carbonate making a contribution to the dimensional stability of samples.

### Dimensional stability under service exposure

**Fig. 8:**
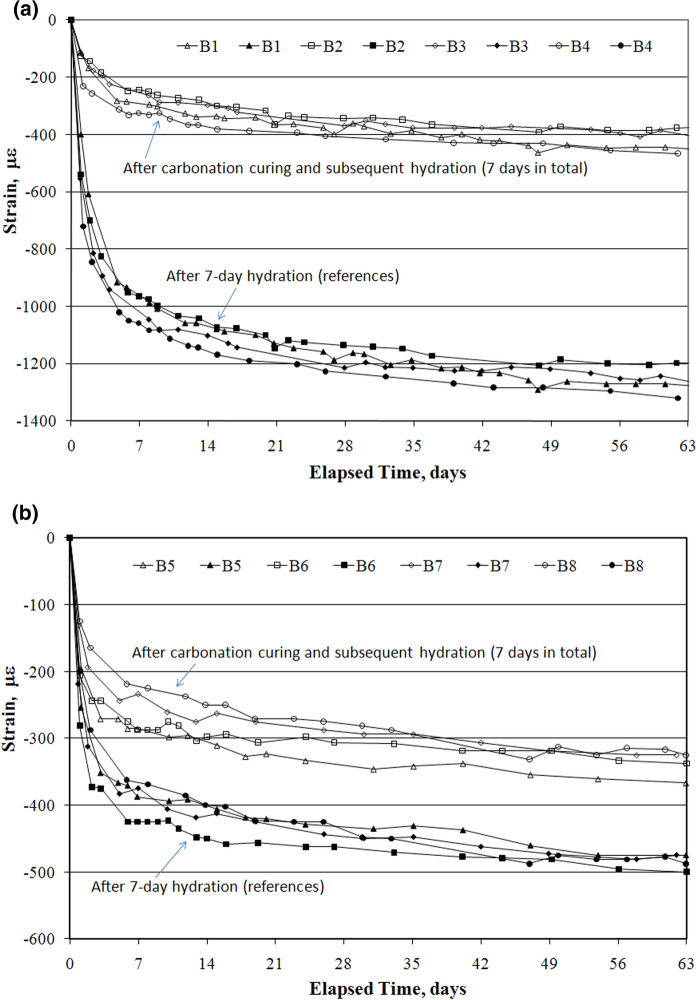
Accelerated weathering carbonation strain curves: **a** Cement paste samples; **b** Concrete samples

Figure 8 presents the strain curves of different samples subject to the 61-day accelerated weathering carbonation test (AWCT) and Table 5 shows the corresponding dimensional stability results. It was clear that both early carbonated paste and concrete samples had significantly improved the resistance to volume change due to weathering carbonation in service exposure. In Fig. 8a, the strain curves of reference hydrated paste samples experienced a rapid increase in shrinkage than those of carbonated samples from the beginning of AWCT before stabilizing to reach a steady-state with an average strain of − 419 με for carbonated samples and of − 1258 με for hydrated samples. The difference of shrinkage between hydrated and carbonated paste was three times on account of carbonation curing. Shown in Table 5, the total strain was defined as the sum of strain values after early-age carbonation curing and after subsequent accelerated weathering carbonation test (AWCT). The final deformations of the pastes were in the form of shrinkage. It was considered as the dimensional stability of sample during curing and in service life. Less total strain indicated more resistance to volume change and less chance to shrinkage cracking. After 61-day weathering carbonation exposure, the total strain recorded in the hydrated paste sample was − 1258 με. This was typical carbonation shrinkage. The larger negative strain recorded in the hydrated sample was attributed to the weathering carbonation of hydration products, mainly Ca(OH)_2_ and C–S–H. By comparing the total strain of hydrated paste (− 1258 με) with those of B1, B2, B3 and B4 (− 751 με in average), it was concluded that early carbonation, preset treatment and increasing carbonation duration could reduce total shrinkage strain of sample owing to the carbonate precipitation during carbonation curing. Similar to the strain curves of cement paste, early carbonated concrete had shown 30% reduction in shrinkage in comparison to reference hydrated concrete during weathering carbonation, reading an average of − 337 με versus − 482 με. Concrete was instinctively more resistant to volume change due to the introduction of aggregates. This was the reason that the difference of curves between early carbonated and normal hydrated concrete in Fig. 8b was not as distinct as that in Fig. 8a. However, during carbonation curing, carbonated concrete experienced some expansions after early-age carbonation curing, which could counteract the shrinkage effect due to the weathering carbonation in service life. Thus, the average total strain of early carbonated concrete was 244 με, only half of that in hydrated concrete, reading − 482 με. After 17-h preset treatment, 18-h carbonation curing and 61-day weathering carbonation exposure, B8 presented the least total strain among early carbonated concrete samples, measuring -145 με. Again, preset treatment and carbonation duration could further contribute to the improved concrete resistance to the volume change due to weathering carbonation. This could decrease the service cracks of concrete products and increase their service lives.

**Table 5:** Strain values of different samples after 61-day accelerated weathering carbonation test (AWCT)

Batch	Carbonation curing	Strain after carbonation curing, με	Strain after AWCT, με	Total strain, με
B1	2hC	− 444	− 446 ± 13	− 890
B2	17hP + 2hC	− 369	− 377 ± 27	− 746
B3	18hC	− 289	− 386 ± 40	− 675
B4	17hP + 18hC	− 225	− 467 ± 9	− 692
B5	2hC	15	− 367 ± 17	− 352
B6	17hP + 2hC	165	− 338 ± 13	− 180
B7	18hC	28	− 325 ± 0	− 297
B8	17hP + 18hC	172	− 317 ± 1	− 145
Hydrated paste	–	–	− 1258 ± 51	− 1258
Hydrated concrete	–	–	− 482 ± 12	− 82

1, 17hP:17-h preset, 2hC: 2-h carbonation; 2, Total strain was the sum of strain values after carbonation curing and after AWCT

Table 6 summarizes the carbon dioxide absorption of samples after 61-day weathering carbonation. It confirmed again that early carbonated samples demonstrated higher resistance to weathering carbonation than normal hydrated samples for both cement paste and concrete. During early-age carbonation curing, carbonates precipitated and densified the sample surface, which could prevent gaseous CO_2_ from further penetration inside. As shown in Table 6, the additional CO_2_ uptakes of carbonation-cured samples before and after weathering carbonation exposure varied from 0.98 to 4.88%, which was much smaller than those of normal hydration samples, ranging the values between 8.7 and 12.88%. More carbon dioxide absorption during weathering carbonation implied more intensive carbonation reactions, thereby producing more carbonation shrinkage and leading to a higher potential for structural cracking.

**Table 6:** Carbon dioxide absorption after 61-day weathering carbonation

Batch	CO_2_ Content by carbon analyzer (slab), %					
	Carbonated samples			Hydrated samples		
	Before	After	Diff	Before	After	Diff
B1	9.52	11.50	1.98	0.52	10.40	9.88
B2	9.24	10.94	1.7	0.52	10.00	9.48
B3	10.89	12.15	1.26	0.52	10.51	9.99
B4	12.75	14.20	1.45	0.52	10.39	9.87
B5	15.22	20.10	4.88	7.22	18.46	11.24
B6	16.10	19.56	3.46	8.36	20.82	12.46
B7	20.39	22.70	2.31	7.52	20.4	12.88
B8	20.11	21.09	0.98	9.25	17.95	8.70

*Before* Before 61-day weathering carbonation, *After* After 61-day weathering carbonation, *Diff* Difference (CO_2_ absorption due to weathering carbonation)

### Compressive strength

#### After early-age carbonation curing

**Fig. 9:**
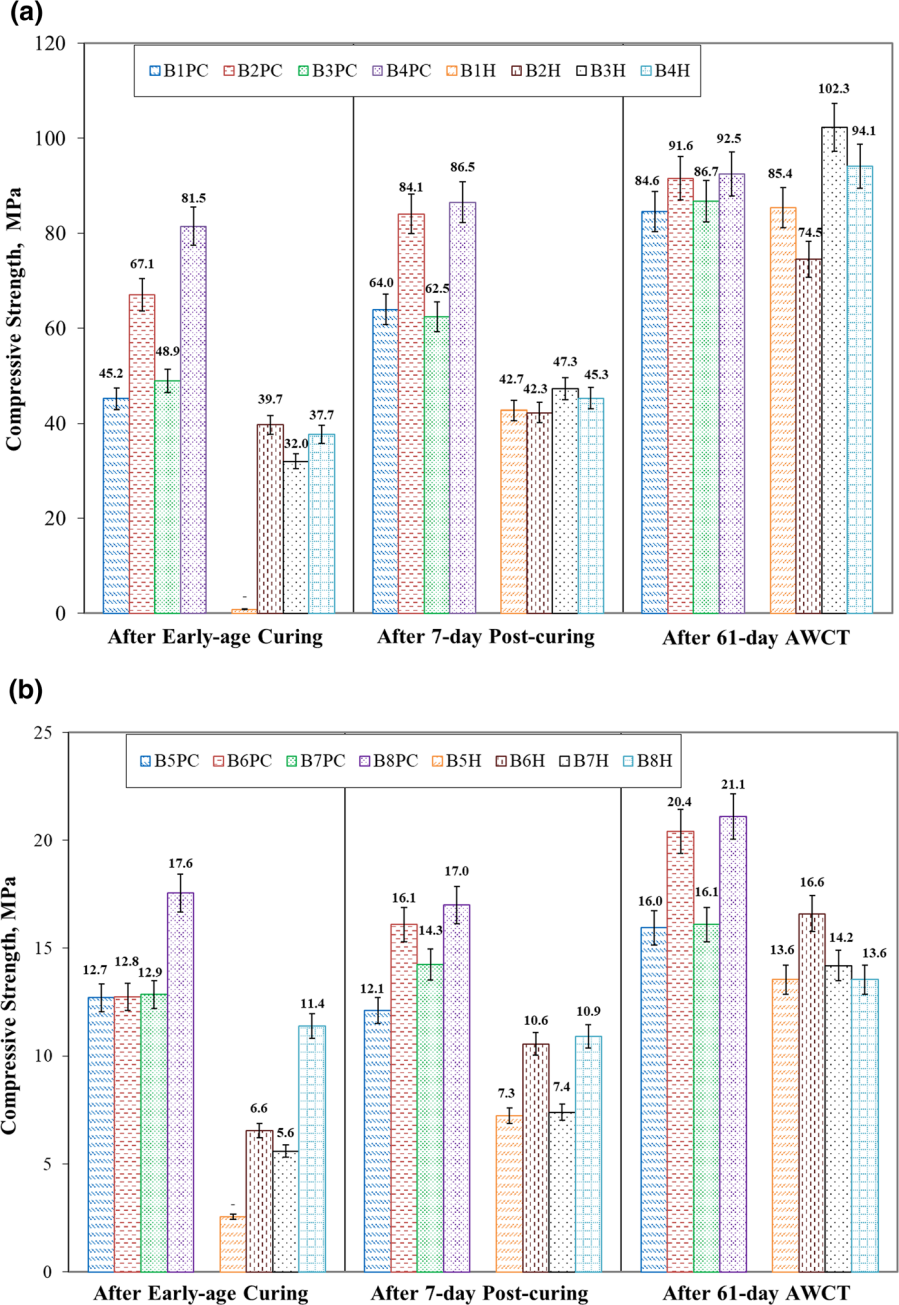
Compressive strength of cement pastes and concretes (PC—Pre-carbonated samples, H—Hydrated samples): **a** Carbonation cured and hydration cured cement pastes; **b** Carbonation cured and hydration cured concretes

During early-age carbonation curing, all carbonated samples experienced volume change due to the carbonate precipitation, specifically resulting in shrinkage in paste while expansion in concrete. It was necessary to investigate the effect of volume change on the mechanical property. Figure 9 summarizes the compressive strength of different samples. It was obvious that all carbonated samples including paste and concrete presented higher compressive strength than their hydration counterparts. Carbonation, preset treatment and increasing carbonation duration could have a significant influence on the strength gain of carbonated samples. After 2-h carbonation, pre-carbonated paste B1PC had the strength of 45.2 MPa while there was no strength in the hydrated paste B1H. Even though increasing carbonation duration to 18 h in B3PC seemed to have limited strength gain than 2-h carbonation in B2PC, its combined effect with 17-h preset treatment dramatically enhanced the strength up to 81.5 MPa in B4PC, which was as twice as that in B4H. It seemed that the carbonate precipitation in the carbonated paste sample not only reduced the hydration shrinkage but also improve the strength.

All carbonated concrete samples experienced small expansions after carbonation curing due to the limited spaces for carbonate precipitation. However, this carbonate-precipitation-induced expansion still contributed to the early strength gain. For the 2-h carbonation of fresh carbonated concrete sample B5PC, its strength was 12.7 MPa in comparison to 2.6 MPa in that of the parallel hydration reference. When increasing immediate carbonation duration to 18-h in B7PC and including 17-h preset treatment before 2-h carbonation in B6PC, both pre-carbonated samples demonstrated much higher strength values than their hydration references. Similar to cement paste, combing 17-h preset treatment and 18-h carbonation in B8PC could have the highest strength after carbonation curing, measuring 17.6 MPa. 1.5 times than its conventionally hydrated samples (11.4 MPa). It turned out that the carbonate precipitation in early carbonated concrete samples could densify the concrete surface making a contribution to the strength development and the long-term durability performance.

#### After 7-day post-curing

After early-age carbonation curing, both carbonated and hydrated samples were kept in a sealed container with relative humidity above 90% to facilitate further hydration, which was vital for the strength and durability development of carbonation-cured cement-based products [31]. This meant the compressive strength herein was tested at 7-day after casting. The result is also shown in Fig. 9. Again, the compressive strengths of all early carbonated samples including paste and concrete were superior than those of normal hydration references. Hydrated paste samples after 7-day had an overall average of 44.4 MPa, which was much smaller than that of carbonated paste sample, calculating 74.3 MPa. Combing the effect of 17-h preset treatment and 18-h carbonation (B4PC) still possessed the highest strength. The strength comparison within carbonated paste samples after 7-day post curing was similar to that right after carbonation curing. Carbonation, preset treatment and longer carbonation duration as 18-h still played an important role in developing strength at subsequent hydration.

Higher strengths still existed in carbonated concrete samples than in normal hydration concretes at 7-day post curing. 2-h carbonation, 17-h presetting plus 2-h carbonation and 18-h carbonation improved the strength from each corresponding counterpart (hydration reference). As was the case for cement paste, the highest strength of concrete sample by carbonation was exhibited by combing preset treatment and longer carbonation curing (B8PC). This indicated that the volume change occurred during carbonation curing did not hinder the subsequent hydration of both paste and concrete samples to further enhance the strength.

#### After 61-day accelerated weathering carbonation

After 61-day weathering carbonation exposure, the volume change of both cement paste and concrete samples ended up with shrinkage. It was essential to examine whether this weathering carbonation shrinkage could affect the strength of samples. Interestingly, it turned out that weathering carbonation could promote later strength for both early carbonated and normal hydrated samples. The strength of samples after weathering carbonation is also summarized in Fig. 9. Although carbonated paste samples had an average of shrinkage strain of − 419 με after 61-day weathering carbonation exposure, they still possessed average strength of 88.9 MPa, comparable to the reading of 89.1 MPa in hydrated samples with a strain of − 1258 με. There were some increases between the strength values of carbonated paste samples with various carbonation curing treatments. Immediately carbonated samples under 2-h or 18-h carbonation had a significantly larger increase in strength of 22.5 MPa on average after AWCT. Paste subject to the combination of 17-h preset treatment and 18-h carbonation still had the highest strength among carbonated pastes (92.5 MPa). Nevertheless, a huge improvement in strength was found in the hydrated paste sample but with considerable variation between hydrated batches after weathering carbonation exposure. It appeared that weathering carbonation of hydration products including C–S–H and calcium hydrate could enhance the sample strength to some extent but also generate shrinkage, which could possibly lead to the occurrence of microcracks. The normal hydrated paste than the early carbonated paste seemed to be more sensitive to weathering carbonation since the latter had a denser surface structure owing to the early carbonation curing. This was consistent with Table 6, in which carbonated paste presented much less carbon dioxide absorption than normal hydrated paste after 61-day weathering carbonation.

Figure 9 displays the compressive strength results of carbonated concrete samples after 61-day weathering carbonation exposure, through which all carbonated concrete samples experienced strength gain. A combination of preset treatment and increased carbonation duration still presented the highest strength. The normal hydrated concrete samples also underwent strength increase after weathering carbonation exposure with an average value of 14.5 MPa, which was smaller than that of pre-carbonated concrete samples recording 18.4 MPa.

It was found that within a certain strain range, there was no obvious correlation between strength development and volume change. Specifically, on the one hand, the early carbonated sample had improved strength with shrinkage for paste while with expansion for concrete after early-age carbonation curing. On the other hand, all samples shrank with enhanced strength after 61-day weathering carbonation exposure.

## Conclusions

The volume change of cement paste and concrete subject to early-age carbonation curing and accelerated weathering carbonation was investigated. It was a holistic study to examine the volume change of cementitious materials due to carbonation at different ages. The in-situ strain measurement indicated that dimensional change of both cement paste and concrete in an early-age carbonation curing process started with a rapid initial shrinkage in the first minute right after gas injection, and then followed by an expansion in ten minutes and a secondary shrinkage in the subsequent reaction. Representing the absolute volume change and possibly leading to shrinkage cracking in restraint conditions, the secondary shrinkage was significant in pastes while negligible in concrete. The final deformation in concrete after carbonation curing was expansion, which was beneficial for structural integrity. Therefore, the concrete seemed to be less vulnerable to carbonation-induced shrinkage in earl-age carbonation curing.

Carbonation duration and preset treatment by 17-h hydration could have an influence on the volume change of cement-based materials. Specifically, a longer carbonation duration could increase the relative expansion in cement paste and concrete thus reducing the overall shrinkage after carbonation curing. Preset treatment before carbonation caused more absolute volume change for cement paste but not for concrete. Because of aggregates, concrete presented less volume change than paste in an overall comparison. XRD and SEM results demonstrated that the carbonate precipitation produced by early-age carbonation curing was the primary reason for the relative expansion of samples improving their resistance to volume change.

Even though higher strength was found in all samples subject to weathering carbonation exposure of 61-day, it caused more carbonation shrinkage in normal hydrated than in early carbonated paste and concrete samples as a result of carbonation of hydration products. The carbonation-cured cement pastes and concretes were more resistant to weathering carbonation in service due to the carbonate precipitation and the consumption of Ca(OH)_2_ during early-age carbonation curing.
